# Coffee intake and decreased amyloid pathology in human brain

**DOI:** 10.1038/s41398-019-0604-5

**Published:** 2019-10-22

**Authors:** Jee Wook Kim, Min Soo Byun, Dahyun Yi, Jun Ho Lee, So Yeon Jeon, Gijung Jung, Han Na Lee, Bo Kyung Sohn, Jun-Young Lee, Yu Kyeong Kim, Seong A Shin, Chul-Ho Sohn, Dong Young Lee, for the KBASE Research Group

**Affiliations:** 1 Department of Neuropsychiatry, Hallym University Dongtan Sacred Heart Hospital, 7 Keunjaebong-Gil, Hwaseong, Gyeonggi 18450 Republic of Korea; 2 Department of Psychiatry, Hallym University College of Medicine, Chuncheon, Gangwon 24252 Republic of Korea; 3 Institute of Human Behavioral Medicine, Medical Research Center Seoul National University, Seoul, 03080 Republic of Korea; 4 Department of Neuropsychiatry, Seoul National University Hospital, Seoul, 03080 Republic of Korea; 5 Department of Psychiatry, Sanggye Paik Hospital, Inje University College of Medicine, Seoul, 01757 Republic of Korea; 6 Department of Neuropsychiatry, SMG-SNU Boramae Medical Center, Seoul, 07061 Republic of Korea; 7 Department of Psychiatry, Seoul National University College of Medicine, Seoul, 03080 Republic of Korea; 8 Department of Nuclear Medicine, SMG-SNU Boramae Medical Center, Seoul, 07061 Republic of Korea; 9 Department of Radiology, Seoul National University College of Medicine, Seoul, 03080 Republic of Korea

**Keywords:** Molecular neuroscience, Psychiatric disorders

## Abstract

Several epidemiological and preclinical studies supported the protective effect of coffee on Alzheimer’s disease (AD). However, it is still unknown whether coffee is specifically related with reduced brain AD pathologies in human. Hence, this study aims to investigate relationships between coffee intake and in vivo AD pathologies, including cerebral beta-amyloid (Aβ) deposition, the neurodegeneration of AD-signature regions, and cerebral white matter hyperintensities (WMH). A total of 411 non-demented older adults were included. Participants underwent comprehensive clinical assessment and multimodal neuroimaging including [^11^C] Pittsburgh compound B-positron emission tomography (PET), [^18^F] fluorodeoxyglucose PET, and magnetic resonance imaging scans. Lifetime and current coffee intake were categorized as follows: no coffee or <2 cups/day (reference category) and ≥2 cups/day (higher coffee intake). Lifetime coffee intake of ≥2 cups/day was significantly associated with a lower Aβ positivity compared to coffee intake of <2 cups/day, even after controlling for potential confounders. In contrast, neither lifetime nor current coffee intake was not related to hypometabolism, atrophy of AD-signature region, and WMH volume. The findings suggest that higher lifetime coffee intake may contribute to lowering the risk of AD or related cognitive decline by reducing pathological cerebral amyloid deposition.

## Introduction

Coffee is one of the most popularly consumed beverages in the world and a high proportion of adults drink coffee daily^1^. Coffee contains hundreds of bioactive compounds, including caffeine, chlorogenic acid, polyphenols, and small amounts of minerals and vitamins, some of which are known to have positive effects on health^2^. Many epidemiological studies suggest that coffee has beneficial effects on various medical conditions, including stroke^3^, heart failure^4^, cancers^5^, diabetes^6^, suicide^7^, Parkinson’s disease^8^, and mortality^9^.

Several epidemiological studies also supported the protective effect of coffee on Alzheimer’s disease (AD)^10–12^ and cognitive decline^13–15^. Nevertheless, there is limited information available on the neuropathological evidences that support the protective effects of coffee on AD and related cognitive decline in humans. Although a preclinical study of aged transgenic AD mice reported that caffeine, a major component of coffee, decreases brain beta-amyloid (Aβ) levels^16–18^, it is still unknown whether coffee is specifically related with reduced brain AD pathologies, including Aβ deposition and regional neurodegenerations in human.

Therefore, we investigate relationships between coffee intake and in vivo AD biomarkers on multimodal brain imaging, including cerebral Aβ deposition, AD-signature region cerebral glucose metabolism (AD-CM), AD-signature region cortical thickness (AD-CT), and cerebral white matter hyperintensities (WMH) in non-demented older adults.

## Methods

### Participants

This study was part of the Korean Brain Aging Study for Early Diagnosis and Prediction of Alzheimer’s Disease (KBASE), which is an ongoing prospective cohort study that begun in 2014^19^. As of February 2017, 411 individuals [282 cognitively normal (CN) adults, and 129 adults with mild cognitive impairment (MCI)], between 55 and 90 years of age were enrolled in the study.

The CN group consisted of participants with a Clinical Dementia Rating (CDR)^20^ score of 0 and no diagnosis of MCI or dementia. All participants with MCI met the current consensus criteria for amnestic MCI, including: (1) memory complaints confirmed by an informant; (2) objective memory impairments; (3) preservation of global cognitive function; (4) independence in functional activities; and (5) no dementia. Regarding Criterion 2, the age-, education-, and gender-adjusted *z*-score was <−1.0 for at least one of four episodic memory tests: Word List Memory, Word List Recall, Word List Recognition, and Constructional Recall tests; these are included in the Korean version of the Consortium to Establish a Registry for Alzheimer’s Disease (CERAD-K) neuropsychological battery^21^. All MCI individuals had a CDR score of 0.5. The exclusion criteria were as follows: (1) presence of a major psychiatric illness; (2) significant neurological or medical condition or comorbidity that could affect mental functioning; (3) contraindications for an magnetic resonance imaging (MRI) scan (e.g., pacemaker or claustrophobia); (4) illiteracy; (5) the presence of significant visual/hearing difficulties and/or severe communication or behavioral problems that would make clinical examinations or brain scans difficult; (6) pregnant or lactation; (7) use of an investigational drug; and (8) drinking tea extract regularly. The Institutional Review Board of Seoul National University Hospital and the SMG-SNU Boramae Medical Center in South Korea approved the present study, and all subjects provided written informed consent prior to participation. More detailed information on recruitment of the KBASE cohort is described in our previous report^19^.

### Clinical and neuropsychological assessments

All participants were administered standardized clinical assessments by trained board-certified psychiatrists based on the KBASE clinical assessment protocol which incorporated the CERAD-K clinical assessment^19^, which incorporates the CERAD-K^22^. All subjects were also given a comprehensive neuropsychological assessment battery, administered by a clinical neuropsychologist or trained psychometrists according to a standardized protocol incorporating the CERAD-K neuropsychological battery^21^. Details on full assessment battery were described previously^19^.

### Assessment of coffee intake

All participants were systematically assessed by trained nurses to determine coffee intake. Specifically, the amount of coffee intake (cups/day) for each participant were assessed for the past one year (i.e., current) and overall lifetime. Previous epidemiologic studies on the effect of coffee intake^10,12,23^ showed that there was a clear difference in the risk of overall or AD dementia between “<2 cups/day (no or lower drinker)” and “≥2 cups/day (higher drinker)” group. Based on the findings, we categorized the participant into the two group, and tried to test the hypothesis that there is a difference in AD pathology between the two.

### Assessment of potential confounders

Coffee intake may be influenced by various other conditions. Therefore, all participants were systematically evaluated about potential confounders, such as lifetime cognitive activity (LCA), occupational complexity, annual income, vascular risk, depression, smoking, and alcohol intake.

Cognitive activity participation frequency was measured by 39-item structured questionnaires^24,25^. The details of the measurement of cognitive activity are described in our previous report^26^. Item scores were averaged to yield separate values for each age period. We then calculated the composite score of LCA to use in the subsequent analysis which was an average of all 4-epoch means. With regard to occupational complexity, we considered only the longest-held occupation and then classified into four levels based on the skill levels described in International Standard Classification of Occupations (http://www.ilo.org/public/english/bureau/stat/isco/). Occupations typically involve simple and routine physical or manual tasks at skill level 1, the performance of tasks, such as operating machinery and electronic equipment; driving vehicles; maintenance and repair of electrical and mechanical equipment; and manipulation, ordering and storage of information at skill level 2, the performance of complex technical and practical tasks that require complex problem solving, reasoning, and decision making in a specialized field at skill level 3, and the performance of tasks that require complex problem-solving, decision-making, and creativity based on an extensive body of theoretical and factual knowledge in a specialized field at skill level 4. Information about occupation was obtained from self-report by the participants and confirmed by reliable informants. Annual income was evaluated and categorized into three groups (below the minimum cost of living (MCL), more than MCL but below twice the MCL, twice the MCL or more (http://www.law.go.kr). The MCL was determined according to the administrative rule published by the Ministry of Health and Welfare, Republic of Korea in November 2012. The MCL was 572,168 Korea Won (KRW) for single-person household and added 286,840 KRW for each additional housemate. The comorbidity rates of vascular risk factors were assessed by interviews of participants and their reliable informants; a vascular risk score (VRS) was calculated based on the number of vascular risk factors present and reported as a percentage^27^. To acquire accurate information, reliable informants were interviewed, and medical records were reviewed. The Geriatric Depression Scale (GDS)^28^ was used to measure the severity of depressive symptoms. Smoking status (never/former/smoker) and alcohol intake status (never/former/drinker) were evaluated through nurse interview. Blood samples were also obtained via venipuncture, genomic DNA was extracted from whole blood and apolipoprotein E (APOE) genotyping was performed as described previously^29^. APOE ε4 (APOE4) positivity was defined as the presence of at least one ε4 allele was present.

### Measurement of cerebral Aβ deposition

All participants underwent simultaneous three-dimensional [^11^C] Pittsburg compound B (PiB)-positron emission tomography (PET) and T1-weighted MRI scans using a 3.0 T Biograph mMR (PET-MR) scanner (Siemens; Washington DC, WC, USA) according to the manufacturer’s guidelines. The details of PiB-PET acquisition and preprocessing were described in our previous report^30^. An AAL algorithm and a region-combining method^31^ were applied to determine the regions of interest (ROIs) for characterization of PiB retention levels in the frontal, lateral parietal, posterior cingulate-precuneus, and lateral temporal regions. The standardized uptake value ratio (SUVR) values for each ROI were calculated by dividing the mean value for all voxels within each ROI by the mean cerebellar uptake value on the same image. Each participant was classified as Aβ positive (Aβ+) if the SUVR value was >1.4 in at least one of the four ROIs^31,32^. Considering the bimodal distribution of our PiB data, only Aβ positivity was used as an outcome variable^33,34^.

### Measurement of AD-CM

All subjects underwent [^18^F] fluorodeoxyglucose (FDG)-PET imaging using the above-described PET-MR machine. The details of FDG-PET acquisition and preprocessing were described in our previous report^30^. AD-signature FDG ROIs that are sensitive to the changes associated with AD, such as the angular gyri, posterior cingulate cortex, and inferior temporal gyri^32^, were determined. AD-CM was defined as the voxel-weighted mean SUVR extracted from the AD-signature FDG ROIs.

### Measurement of AD-CT

All T1-weighted images were acquired in the sagittal orientation using the above-described 3.0 T PET-MR machine. MR image acquisition and preprocessing were described in our previous report^30^. AD-CT was defined as the mean cortical thickness values obtained from AD-signature regions including the entorhinal, inferior temporal, middle temporal, and fusiform gyrus, as described previously^32^.

### Measurement of WMH

All participants underwent MRI scans with fluid attenuated inversion recovery using the abovementioned 3.0 T PET-MR scanner in a validated automatic procedure that has previously been reported^35^. The details of the volume measurement of cerebral WMH were previously described^36^.

### Statistical analysis

We first compared demographic variables, other potential confounders [APOE4, clinical diagnosis (CN vs. MCI), LCA score, occupational complexity, annual income status, VRS, GDS score, smoking status, and alcohol intake status] for the relationship between coffee intake and AD biomarkers, and AD imaging biomarkers between lifetime coffee intake categories (<2 cups/day and ≥2 cups/day) by *t* test or *χ*^2^ test as appropriate. In order to explore the relationship between lifetime coffee intake amount and potential confounders, we performed Spearman correlation analyses. To examine the relationships between lifetime (or current) coffee intake category and neuroimaging parameters, multivariate logistic or linear regression analyses were performed as appropriate. In these analyses, “<2 cups/day” category was used as a reference. Three models were tested for controlling the covariates stepwisely. The first model included age, gender, education, APOE4, clinical diagnosis as covariates; the second model included covariates in the first model plus LCA score, occupational complexity, annual income status, VRS, GDS score, smoking status, and alcohol intake status; and third model included covariates in the second model plus the duration of coffee intake and the age of first coffee intake. To reduce false positive error due to multiple testing, we applied Bonferroni correction. Actually, *p* < 0.00625 (=0.05/8) was used as the threshold for statistical significance for each analysis considering 4 biomarkers and 2 time periods.

For the AD neuroimaging biomarker with significant association with coffee intake in above analyses, additional exploratory analyses were performed. First, to explore whether there are any brain regional specificity in regard of the relationship between lifetime coffee intake and the biomarker, the same analysis was done for each of the four ROI (i.e., the frontal, lateral parietal, posterior cingulate-precuneus, and lateral temporal region). Second, in order to investigate the modulating effects of the potential confounders (i.e., age, gender, education, APOE4, clinical diagnosis, LCA score, occupational complexity, annual income status, VRS, GDS score, smoking status, and alcohol intake status) on the relationships between coffee intake and the biomarker, we performed the same analysis including two-way interaction term between coffee intake and any one of the confounders, as well as coffee intake itself, as an independent variable. We additionally examined the three-way interaction between lifetime coffee intake and any two of age, education, gender, and APOE4 on the relationship between coffee intake and the biomarker. Third, to explore the dose-effect relationship between overall amount of coffee intake and the biomarker, the same analysis including the total amount of lifetime coffee intake (=duration of coffee intake × cups of coffee intake/day) as an independent variable instead of coffee intake category (lower vs. higher) were performed. For similar purpose, we also compared the AD biomarker among four coffee intake categories (i.e., 0 or <1 cups/day, 1≤ and <2 cups/day, 2≤ and <3 cups/day, and 3≤ cups/day) instead of the dichotomous categories by using *χ*^2^ test. For these exploratory analyses, *p* < 0.05 was served as a statistical threshold. All statistical analyses were performed using IBM SPSS Statistics 24 software (IBM Corp., Armonk, NY, USA).

## Results

### Participant characteristics

The demographic and clinical characteristics of the participants are presented by the categories of lifetime coffee intake in Table 1. Of the 411 participants, 269 were no or lower coffee drinkers (<2 cups/day) and 142 were higher coffee drinkers (≥2 cups/day). There were significant differences of sex, education, duration of coffee intake, age of first coffee intake, LCA score, occupational complexity, smoking status, alcohol drinking status, and Aβ positivity between the two lifetime coffee intake groups. Correlations of lifetime coffee intake amount with potential confounders for the relationship between coffee intake and AD biomarkers were also presented in Supplementary Table 1.

**Table 1:** Participant characteristics^a^

Characteristic	Coffee intake amount, lifetime			*t* or *χ*^2^	*p* Value
	<2 cups/day	≥2 cups/day	Total		
*n*	269	142	411		
Age, y	71.06 (7.73)	69.67 (8.43)	70.58 (8.00)	1.675	0.095
Female, no. (%)	175 (65.06)	57 (40.04)	232 (56.45)	23.467	<0.001
Education, y	10.56 (4.90)	12.27 (4.49)	11.15 (4.82)	−3.574	<0.001
MMSE	25.26 (3.41)	25.96 (3.34)	25.50 (3.40)	−2.007	0.045
APOE4 positivity, no. (%)	61 (22.76)	35 (24.65)	96 (23.41)	0.184	0.668
Clinical diagnosis, CN, no. (%)	183 (68.03)	99 (69.72)	282 (68.61)	0.123	0.726
Duration of coffee intake, y	27.61 (19.06)	34.12 (15.06)	25.93 (18.73)	−6.784	<0.001
Age of first coffee intake, y	41.17 (17.94)	34.03 (14.54)	38.31 (17.01)	3.940	<0.001
Cognitive activity					
Childhood score	2.00 (0.64)	2.06 (0.58)	2.02 (0.62)	−0.892	0.373
Adulthood score	2.29 (0.90)	2.46 (0.84)	2.35 (0.86)	−1.800	0.073
Midlife score	2.24 (0.84)	2.44 (0.79)	2.31 (0.83)	−2.338	0.020
Current score	2.37 (0.69)	2.52 (0.71)	2.42 (0.70)	−1.934	0.054
Lifetime composite score	2.23 (0.67)	2.37 (0.59)	2.27 (0.64)	−2.113	0.035
Occupational complexity, no. (%)				11.571	0.021
None	59 (22.01)	16 (11.27)	75 (18.29)		
Skill level 1	20 (7.46)	9 (6.34)	29 (7.07)		
Skill level 2	88 (32.84)	44 (30.99)	132 (30.19)		
Skill level 3	28 (10.45)	26 (18.31)	54 (13.17)		
Skill level 4	73 (27.24)	47 (33.10)	120 (29.27)		
Annual income, no. (%)				2.530	0.282
<MCL	19 (7.06)	16 (11.27)	35 (8.52)		
≥MCL, <2 × MCL	124 (46.10)	58 (40.85)	182 (44.28)		
≥2 × MCL	126 (46.84)	68 (47.89)	194 (47.20)		
VRS	18.77 (15.76)	16.31 (17.47)	17.92 (16.39)	0.148	0.148
GDS score	6.65 (5.95)	6.47 (6.76)	6.59 (6.24)	0.270	0.787
Smoking status, no. (%)				27.087	<0.001
Never	206 (76.58)	73 (51.41)	279 (67.88)		
Former	54 (20.07)	58 (40.85)	112 (27.25)		
Smoker	9 (3.35)	11 (7.75)	20 (4.87)		
Alcohol drink status, no. (%)				12.651	0.002
Never	161 (59.85)	63 (44.37)	224 (54.50)		
Former	25 (9.29)	28 (19.72)	53 (12.90)		
Drinker	83 (30.86)	51 (35.92)	134 (32.60)		
Cerebral Aβ deposition					
Aβ positivity, no. (%)	73 (27.14)	25 (17.61)	98 (23.84)	4.650	0.031
Neurodegeneration					
AD-CM, SUVR	1.40 (0.13)	1.39 (0.12)	1.39 (0.13)	0.462	0.645
AD-CT, mm	2.81 (0.22)	2.80 (0.23)	2.81 (0.22)	0.262	0.794
WMH volume, cm^3^	5.89 (5.56)	6.02 (5.03)	5.94 (5.37)	−0.217	0.828

*APOE4* apolipoprotein ε4, *CN* cognitive normal, *MCL* minimum cost of living, *VRS* vascular risk score, *GDS* Geriatric depression scale, *Aβ* beta-amyloid, *AD* Alzheimer’s disease, *AD-CM* Alzheimer’s disease signature cerebral glucose metabolism, *AD-CT* Alzheimer’s disease signature cortical thickness, *SUVR* standardized uptake value ratio, *WMH* white matter hyperintensities

^a^Unless otherwise indicated, data are expressed as mean (standard deviation)

### Difference of Aβ positivity between high and low coffee intakes

**Fig. 1:**
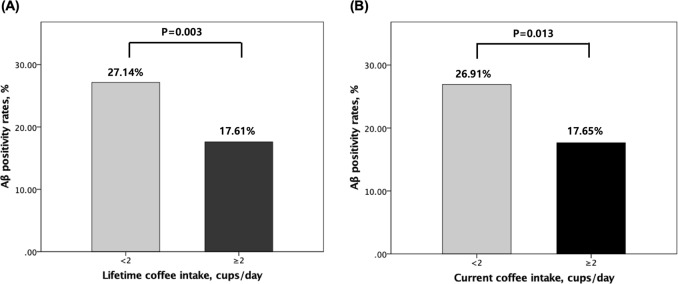
Aβ positivity rates according to the categories of (**a**) lifetime or (**b**) current coffee intake: comparison of Aβ positivity rates between 0 or <2 cups/day and ≥2 cups/day in non-demented older adults. Multivariate logistic regression analyses were performed after controlling for age, gender, education, apolipoprotein ε4, clinical diagnosis, LCA score, occupational complexity, annual income status, VRS, GDS score, smoking status, alcohol intake status, duration of coffee intake and age of first coffee intake. *Aβ* beta-amyloid, LCA lifetime cognitive activity, VRS vascular risk score, GDS geriatric depression scale. The English in this document has been checked by at least two professional editors, both native speakers of English. For a certificate, please see: http://www.textcheck.com/certificate/1uduau

The association between coffee intake and Aβ positivity presented in Table 2 and Fig. 1. Lifetime coffee intake of ≥2 cups/day showed significantly lower Aβ positivity compared to coffee intake of <2 cups/day, regardless of the models. To explore whether there are any brain regional specificity in regard of the relationship between lifetime coffee intake and Aβ positivity, the difference of Aβ positivity between high and low lifetime coffee intakes was tested for each of the four ROI (i.e., the frontal, lateral parietal, posterior cingulate-precuneus, and lateral temporal region). Lifetime coffee intake of ≥2 cups/day showed lower Aβ positivity in all four regions (Table 3). In contrast to lifetime coffee intake, current coffee intake was not related to Aβ positivity regardless of the covariates.

**Table 2:** Results of multiple logistic regression analyses for assessing the relationships of stratified coffee intake with Aβ positivity in non-demented individuals

Coffee intake	Aβ positivity	
	OR (95% CI)	*p* Value
*Model 1* ^a^		
Lifetime		
<2 cup/day	Reference	
≥2 cup/day	0.401 (0.208 to 0.772)	0.006^*^
Current		
<2 cup/day	Reference	
≥2 cup/day	0.453 (0.236 to 0.869)	0.017
*Model 2* ^b^		
Lifetime		
<2 cup/day	Reference	
≥2 cup/day	0.386 (0.197 to 0.757)	0.006^*^
Current		
<2 cup/day	Reference	
≥2 cup/day	0.443 (0.227 to 0.862)	0.017
*Model 3* ^c^		
Lifetime		
<2 cup/day	Reference	
≥2 cup/day	0.334 (0.162 to 0.689)	0.003^*^
Current		
<2 cup/day	Reference	
≥2 cup/day	0.402 (0.197 to 0.822)	0.013

*Aβ* beta-amyloid, *OR* odds ratio, *CI* confidence interval, *APOE4* apolipoprotein ε4, *LCA* lifetime cognitive activity, *VRS* vascular risk score, *GDS* geriatric depression scale

^a^ Adjusted for age, gender, education, apolipoprotein ε4, and clinical diagnosis

^b^ Adjusted for covariates in Model 1 plus, LCA score, occupational complexity, and annual income status, VRS, GDS score, smoking status, and alcohol status

^c^ Adjusted for covariates in Model 2 plus, duration of coffee intake and age of first coffee intake

^*^Statistically significant (*p* < 0.00625)

**Table 3:** Results of multiple logistic regression analyses for assessing the relationship between stratified lifetime coffee intake and subregional Aβ positivity in non-demented individuals

Lifetime coffee intake	Frontal region		PC-PRC region		Lt. parietal region		Lt. temporal region	
	OR (95% CI)	*p*	OR (95% CI)	*p*	OR (95% CI)	*p*	OR (95% CI)	*p*
*Model 1* ^a^								
<2 cup/day	Reference		Reference		Reference		Reference	
≥2 cup/day	0.400 (0.197 to 0.813)	0.011	0.417 (0.218 to 0.798)	0.008	0.393 (0.197 to 0.783)	0.008	0.500 (0.252 to 0.993)	0.048
*Model 2* ^b^								
<2 cup/day	Reference		Reference		Reference		Reference	
≥2 cup/day	0.381 (0.183 to 0.793)	0.010	0.402 (0.206 to 0.783)	0.007	0.370 (0.181 to 0.757)	0.007	0.475 (0.234 to 0.966)	0.040
*Model 3* ^c^								
<2 cup/day	Reference		Reference		Reference		Reference	
≥2 cup/day	0.324 (0.149 to 0.707)	0.005	0.349 (0.170 to 0.713)	0.004	0.285 (0.132 to 0.617)	0.001	0.400 (0.188 to 0.851)	0.017

*Aβ* beta-amyloid, *PC-PRC* posterior cingulate-precuneus, *OR* odds ratio, *CI* confidence interval, *APOE4* apolipoprotein ε4, *LCA* lifetime cognitive activity, *VRS* vascular risk score, *GDS* geriatric depression scale

^a^Adjusted for age, gender, education, apolipoprotein ε4, and clinical diagnosis

^b^Adjusted for covariates in Model 1 plus, LCA score, occupational complexity, annual income status, VRS, GDS score, smoking status, and alcohol status

^c^Adjusted for covariates in Model 2 plus, duration of coffee intake and age of first coffee intake

### Moderating effect of potential confounders on the relationship between lifetime coffee intake and Aβ positivity

Any two-way interaction between lifetime coffee intake and each of age, gender, gender, APOE4, clinical diagnosis, LCA score, occupational complexity, annual income status, VRS, GDS score, smoking status, and alcohol intake status was not significant, indicating that the potential confounders do not moderate the relationship between lifetime coffee intake and Aβ positivity (Supplementary Table 2). We additionally examined the three-way interaction between lifetime coffee intake and any two of age, gender, education, and APOE4 on the relationship between coffee intake and Aβ positivity, but did not find any significant finding.

### Dose–effect relationship between lifetime coffee intake and Aβ positivity

To explore the dose-effect relationship between lifetime coffee intake amount and Aβ positivity further, we compared Aβ positivity rates according to four lifetime coffee intake strata, i.e., 0 or <1 cups/day, 1≤ and <2 cups/day, 2≤ and <3 cups/day, and 3≤ cups/day by using *χ*^2^ test. As shown in Supplementary Fig. 1, there was a significant trend of association between lifetime coffee intake strata and Aβ positivity (*p* = 0.048). Multiple logistic regression analysis also demonstrated that there was a trend toward significance on dose–effect association between the total amount of lifetime coffee intake (=duration of coffee intake × cups of coffee intake/day) and Aβ positivity [OR (95% CI) = 0.991 (0.982–1.001), *p* = 0.067]. As the amount increased, so Aβ-positivity rate decreased (Supplementary Table 3).

### Association of coffee intake with cerebral tau deposition, AD-CM, AD-CT, and WMH

In contrast to the results for Aβ positivity, neither lifetime nor current coffee intake was related with any of AD-CM, AD-CT, and WMH (Table 4).

**Table 4:** Results of multiple linear model analyses for assessing the relationship between stratified coffee intake and AD-CM, AD-CT, or WMH volume in non-demented individuals

Coffee intake	AD-CM		AD-CT		WMH	
	*B* (95% CI)	*p* Value	B (95% CI)	*p* value	*B* (95% CI)	*p* Value
*Model 1* ^a^						
Lifetime						
<2 cup/day	Reference		Reference		Reference	
≥2 cup/day	−0.007 (−0.034 to 0.021)	0.633	0.002 (−0.037 to 0.042)	0.910	0.237 (−0.990 to 1.464)	0.704
Current						
<2 cup/day	Reference		Reference		Reference	
≥2 cup/day	−0.009 (−0.037 to 0.019)	0.540	−0.001 (−0.041 to 0.040)	0.980	0.620 (−0.626 to 1.867)	0.328
*Model 2* ^b^						
Lifetime						
<2 cup/day	Reference		Reference		Reference	
≥2 cup/day	−0.008 (−0.035 to 0.020)	0.580	0.003 (−0.037 to 0.042)	0.888	0.282 (−0.961 to 1.526)	0.655
Current						
<2 cup/day	Reference		Reference		Reference	
≥2 cup/day	−0.013 (−0.040 to 0.015)	0.369	−0.002 (−0.041 to 0.038)	0.935	0.674 (−0.591 to 1.939)	0.295
*Model 3* ^c^						
Lifetime						
<2 cup/day	Reference		Reference		Reference	
≥2 cup/day	0.006 (−0.022 to 0.033)	0.678	0.008 (−0.033 to 0.048)	0.707	0.181 (−1.124 to 1.486)	0.785
Current						
<2 cup/day	Reference		Reference		Reference	
≥2 cup/day	0.001 (−0.027 to 0.029)	0.951	0.004 (−0.038 to 0.045)	0.864	0.612 (−0.714 to 1.938)	0.365

*Aβ* beta-amyloid, *AD-CM* Alzheimer’s disease signature cerebral glucose metabolism, *AD-CT* Alzheimer’s disease signature cortical thickness, *WMH* white matter hyperintensities, *CI* confidence interval, *LCA* lifetime cognitive activity, *GDS* geriatric depression scale, *APOE4* apolipoprotein ε4

^a^Adjusted for age, gender, education, APOE4, and clinical diagnosis

^b^Adjusted for covariates in Model 1 plus, LCA score, occupational complexity, annual income status, vascular risk score, GDS score, smoking status, and alcohol status

^c^Adjusted for covariates in Model 2 plus, duration of coffee intake and age of first coffee intake

## Discussion

The present study found that a lifetime coffee intake of ≥2 cups/day (higher coffee intake) was associated with lower cerebral Aβ positivity rate in non-demented older adults when compared to the coffee intake of <2 cups/day. We did not find any association of coffee intake with regional neurodegeneration and WMH. This is the first study to investigate the association between higher coffee intake and in vivo AD pathologies in human.

The present finding of the relationship between higher coffee intake and a decreased rate of pathological Aβ deposition is in line with results from previous studies using animal models, which indicated that higher caffeine, one of the major ingredients of coffee, intake exerts a protective effect via molecular Aβ-related mechanisms^16–18,37,38^. For example, Arendash et al.^18^ suggested that caffeine protects AD mice against cognitive impairment and reduces brain Aβ production by deactivating the positive-feedback loop from the γ- to β-secretase cleavages on the Aβ protein precursor. The same group also reported that high caffeine intake improves cognitive performance of aged AD mice, but not of aged wild-type mice, with reduced brain Aβ levels, suggesting that the cognitive enhancing effect of caffeine in AD mice is mediated by a decrease in Aβ concentration^16^. Furthermore, Cao et al. reported that caffeine suppresses Aβ levels in the plasma and brain of AD mice^17^ and also suggested that caffeine and other components in coffee may synergize to protect against cognitive decline in AD mice^38^. Moreover, Li et al.^37^ indicated that caffeine suppresses Aβ protein precursor internalization and Aβ generation via adenosine A3 receptor-mediated actions. The present finding also provides a neuropathological explanation for the relationship between higher coffee intake and reduced risk of AD dementia observed in several clinical and epidemiological studies^10–12^. Those studies reported higher coffee drinkers had 31–65% decrease in the risk of AD dementia, which is quite comparable to about 65% decrease of Aβ positivity rate in higher coffee drinkers (27.14%) compared to lower coffee drinkers (17.61%). Furthermore, the relationship between higher coffee intake and lower Aβ positivity was prominent for lifetime coffee intake than for current coffee intake. This suggests that the protective effects of higher coffee intake against Aβ pathology involve the chronic effects associated with prolonged exposure rather than an acute or short-term effect.

In the present study, we did not find any association of coffee intake with regional neurodegeneration and WMH. Although no previous study investigated the relationship between coffee intake and brain metabolism, the Honolulu-Asia Aging Study showed that coffee intake was not associated with generalized brain atrophy and microvascular ischemic lesions^39^, similarly to our findings. In addition, the Health Professional Follow-up Study also showed that chronic coffee or caffeine intake is not associated with a risk of cerebrovascular or cardiovascular disease^40^. Although some previous reports indicated an association between coffee intake and cerebrovascular risk, they examined the acute effect of coffee intake, but not the chronic effect of long-term coffee intake^41,42^. Such a null association between coffee intake and AD-related neurodegeneration or vascular changes indicates that chronic coffee intake has no direct effects on neurodegenerative or cerebrovascular changes through Aβ-independent mechanisms. Given the significant association between higher coffee intake and lower Aβ positivity, the negative finding for AD-related regional neurodegeneration appears related to the long-time delay between pathological Aβ accumulation and Aβ-dependent neurodegeneration^43,44^.

The present study had several limitations that should be considered. First, because this was a cross-sectional study, it is difficult to infer causal relationships from the findings. However, the significant relationship between lifetime coffee intake and amyloid pathology supports the possible causal nature of the relationship. Second, underestimates of coffee intake or retrospective recall bias may have affected the results of lifetime coffee intake in older individuals. However, coffee intake is less prone to misreporting because coffee intake is a long-term habitual behavior. Evaluation for coffee intake is known to be performed with the highest validity and reproducibility^45^. In addition, the current finding between coffee intake and amyloid was significant even after controlling the effect of clinical diagnosis on cognitive status, and the reported frequency of coffee intake was not related with the proportion of MCI (Table 1). Finally, it is unclear which ingredient(s) in coffee acts on Aβ pathology. Although caffeine is among hundreds of bioactive compounds in coffee^46^, it is the most widely studied ingredient against Aβ pathology^16–18^. Other bioactive compounds include chlorogenic acid, polyphenols, small amount of minerals, and vitamin B_3_, which have also been investigated^47–49^. However, it remains controversial whether a single ingredient in coffee is effective against Aβ pathology or whether a combination of ingredients is effective. Therefore, further investigations are needed to clarify which ingredient(s) in coffee are important for reducing Aβ pathology. The comparison between coffee with and without caffeine may give us a clue on the specific effect of caffeine.

In conclusion, the findings of present study suggest that higher lifetime coffee intake is likely to contribute to lowering the risk of AD or related cognitive decline by reducing pathological cerebral amyloid deposition.

## Supplementary information

Supplementary Table1, Supplementary Table 2, Supplementary Table 3

Supplementary Figure 1

## Data Availability

The datasets generated and analyzed during the present study are not publicly available, owing to ethics considerations and privacy restrictions. Data may be obtained from the corresponding author after approval by the Institutional Review Board of the Seoul National University Hospital, South Korea has been sought.

## References

[1] Loftfield E (2016). Coffee drinking is widespread in the United States, but usual intake varies by key demographic and lifestyle factors. J. Nutr..

[2] Spiller MA (1984). The chemical components of coffee. Prog. Clin. Biol. Res..

[3] Larsson SC, Virtamo J, Wolk A (2011). Coffee consumption and risk of stroke in women. Stroke.

[4] Mostofsky E, Rice MS, Levitan EB, Mittleman MA (2012). Habitual coffee consumption and risk of heart failure: a dose-response meta-analysis. Circ. Heart Fail..

[5] Wang A (2016). Coffee and cancer risk: a meta-analysis of prospective observational studies. Sci. Rep..

[6] Akash MS, Rehman K, Chen S (2014). Effects of coffee on type 2 diabetes mellitus. Nutrition.

[7] Lucas M (2014). Coffee, caffeine, and risk of completed suicide: results from three prospective cohorts of American adults. World J. Biol. Psychiatry.

[8] Ross GW (2000). Association of coffee and caffeine intake with the risk of Parkinson disease. J. Am. Med. Assoc..

[9] Ding M (2015). Association of Coffee Consumption With Total and Cause-Specific Mortality in 3 Large Prospective Cohorts. Circulation.

[10] Eskelinen MH, Kivipelto M (2010). Caffeine as a protective factor in dementia and Alzheimer’s disease. J. Alzheimer Dis..

[11] Lindsay J (2002). Risk factors for Alzheimer's disease: a prospective analysis from the Canadian Study of Health and Aging. Am. J. Epidemiol..

[12] Eskelinen MH, Ngandu T, Tuomilehto J, Soininen H, Kivipelto M (2009). Midlife coffee and tea drinking and the risk of late-life dementia: a population-based CAIDE study. J. Alzheimer Dis..

[13] Arab L (2011). Gender differences in tea, coffee, and cognitive decline in the elderly: the Cardiovascular Health Study. J. Alzheimer Dis..

[14] van Gelder BM (2007). Coffee consumption is inversely associated with cognitive decline in elderly European men: the FINE Study. Eur. J. Clin. Nutr..

[15] Vercambre MN, Berr C, Ritchie K, Kang JH (2013). Caffeine and cognitive decline in elderly women at high vascular risk. J. Alzheimer Dis..

[16] Arendash GW (2009). Caffeine reverses cognitive impairment and decreases brain amyloid-beta levels in aged Alzheimer's disease mice. J. Alzheimer Dis..

[17] Cao C (2009). Caffeine suppresses amyloid-beta levels in plasma and brain of Alzheimer's disease transgenic mice. J. Alzheimer Dis..

[18] Arendash GW (2006). Caffeine protects Alzheimer's mice against cognitive impairment and reduces brain beta-amyloid production. Neuroscience.

[19] Byun MS (2017). Korean Brain Aging Study for the Early Diagnosis and Prediction of Alzheimer’s Disease: methodology and baseline sample characteristics. Psychiatry Investig..

[20] Morris JC (1993). The Clinical Dementia Rating (CDR): current version and scoring rules. Neurology.

[21] Lee DY (2004). A normative study of the CERAD neuropsychological assessment battery in the Korean elderly. J. Int. Neuropsychological Soc..

[22] Lee JH (2002). Development of the Korean version of the Consortium to Establish a Registry for Alzheimer's Disease Assessment Packet (CERAD-K): clinical and neuropsychological assessment batteries. J. Gerontol. Ser. B Psychol. Sci. Soc. Sci..

[23] Driscoll I (2016). Relationships between caffeine intake and risk for probable dementia or global cognitive impairment: the Women’s Health Initiative Memory Study. J. Gerontol. Ser. A Biol. Sci. Med. Sci..

[24] Wilson RS (2005). Early and late life cognitive activity and cognitive systems in old age. J. Int. Neuropsychol. Soc..

[25] Wilson RS, Scherr PA, Schneider JA, Tang Y, Bennett DA (2007). Relation of cognitive activity to risk of developing Alzheimer disease. Neurology.

[26] Ko K (2018). Early-life cognitive activity is related to reduced neurodegeneration in Alzheimer signature regions in late life. Front. Aging Neurosci..

[27] DeCarli C (2004). Memory impairment, but not cerebrovascular disease, predicts progression of MCI to dementia. Neurology.

[28] Kim JY (2008). Standardization of the korean version of the geriatric depression scale: reliability, validity, and factor structure. Psychiatry Investig..

[29] Wenham PR, Price WH, Blandell G (1991). Apolipoprotein E genotyping by one-stage PCR. Lancet.

[30] Park JC (2019). Plasma tau/amyloid-beta1–42 ratio predicts brain tau deposition and neurodegeneration in Alzheimer's disease. Brain.

[31] Reiman EM (2009). Fibrillar amyloid-beta burden in cognitively normal people at 3 levels of genetic risk for Alzheimer's disease. Proc. Natl Acad. Sci. USA.

[32] Jack CR (2014). Age-specific population frequencies of cerebral beta-amyloidosis and neurodegeneration among people with normal cognitive function aged 50–89 years: a cross-sectional study. Lancet Neurol..

[33] Klunk WE (2011). Amyloid imaging as a biomarker for cerebral beta-amyloidosis and risk prediction for Alzheimer dementia. Neurobiol. Aging.

[34] Gottesman RF (2017). Association between midlife vascular risk factors and estimated brain amyloid deposition. J. Am. Med. Assoc..

[35] Tsai JZ (2014). Automated segmentation and quantification of white matter hyperintensities in acute ischemic stroke patients with cerebral infarction. PloS ONE.

[36] Moon SW (2019). The ankle-brachial index is associated with cerebral beta-amyloid deposition in cognitively normal older adults. J. Gerontol. Ser. A Biol. Sci. Med. Sci..

[37] Li S (2015). Caffeine, through adenosine a3 receptor-mediated actions, suppresses amyloid-beta protein precursor internalization and amyloid-beta generation. J. Alzheimer Dis..

[38] Cao C (2011). Caffeine synergizes with another coffee component to increase plasma GCSF: linkage to cognitive benefits in Alzheimer's mice. J. Alzheimer Dis..

[39] Gelber RP, Petrovitch H, Masaki KH, Ross GW, White LR (2011). Coffee intake in midlife and risk of dementia and its neuropathologic correlates. J. Alzheimer Dis..

[40] Grobbee DE (1990). Coffee, caffeine, and cardiovascular disease in men. N. Engl. J. Med..

[41] Mostofsky E, Schlaug G, Mukamal KJ, Rosamond WD, Mittleman MA (2010). Coffee and acute ischemic stroke onset: the Stroke Onset Study. Neurology.

[42] Ritchie K (2010). Caffeine, cognitive functioning, and white matter lesions in the elderly: establishing causality from epidemiological evidence. J. Alzheimer Dis..

[43] Jack CR (2010). Hypothetical model of dynamic biomarkers of the Alzheimer's pathological cascade. Lancet Neurol..

[44] Jack CR (2013). Tracking pathophysiological processes in Alzheimer's disease: an updated hypothetical model of dynamic biomarkers. Lancet Neurol..

[45] Watson EJ, Kohler M, Banks S, Coates AM (2017). Validation and reproducibility of an Australian caffeine food frequency questionnaire. Int J. Food Sci. Nutr..

[46] Nuhu AA (2014). Bioactive micronutrients in coffee: recent analytical approaches for characterization and quantification. ISRN Nutr..

[47] Fukuyama Kazuya, Kakio Shota, Nakazawa Yosuke, Kobata Kenji, Funakoshi-Tago Megumi, Suzuki Toshiharu, Tamura Hiroomi (2018). Roasted Coffee Reduces β-Amyloid Production by Increasing Proteasomal β-Secretase Degradation in Human Neuroblastoma SH-SY5Y Cells. Molecular Nutrition & Food Research.

[48] Dhouafli Zohra, Cuanalo-Contreras Karina, Hayouni El Akrem, Mays Charles E., Soto Claudio, Moreno-Gonzalez Ines (2018). Inhibition of protein misfolding and aggregation by natural phenolic compounds. Cellular and Molecular Life Sciences.

[49] Turunc Bayrakdar E, Uyanikgil Y, Kanit L, Koylu E, Yalcin A (2014). Nicotinamide treatment reduces the levels of oxidative stress, apoptosis, and PARP-1 activity in Abeta(1–42)-induced rat model of Alzheimer's disease. Free Radic. Res..

