# Supplementary material for: Coffee intake and decreased amyloid pathology in human brain
Source: Transl Psychiatry. 2019 Oct 22;9:270. doi: 10.1038/s41398-019-0604-5 (PMC6805864; doi:10.1038/s41398-019-0604-5)
Supplement: Supplementary file 1 — Supplementary Table1, Supplementary Table 2, Supplementary Table 3 [file 41398_2019_604_MOESM1_ESM.docx]

**Supplementary information content**

**Supplementary Table 1** Correlation between lifetime coffee intake amount and potential covariates

**Supplementary Table 2** Results of the multiple logistic regression analyses including the interaction term between lifetime coffee intake and potential covariate(s) predicting Aβ positivity

**Supplementary Table 3** Results of multiple logistic regression analyses for assessing the relationships of overall amount of coffee intake with Aβ positivity in non-demented individuals

**Supplementary Figure 1** Aβ positivity rates according to the categories of lifetime coffee intake amount in quartiles (0 or < 1 cup/day vs. 1≤ and <2 cups/day vs. 2≤and <3cups/day vs. ≥3 cups/day) in non-demented older adults.

Multivariate logistic regression analyses were performed after controlling for age, gender, education, apolipoprotein ε4, clinical diagnosis, LCA score, occupational complexity, annual income status, VRS, GDS score, smoking status, alcohol intake status, duration of coffee intake and age of first coffee intake.

*Abbreviations: Aβ* beta-amyloid, *LCA* lifetime cognitive activity, *VRS* vascular risk score, *GDS* geriatric depression scale.

| **Supplementary Table 1** Correlation between lifetime coffee intake amount and potential covariates | | | |  |
| --- | --- | --- | --- | --- |
|  | | Lifetime coffee intake | |  |
|  | | Spearman correlation coefficient | *P value* |  |
| Age | | -0.103 | 0.036 |  |
| Education | | 0.166 | 0.001 |  |
| LCA score | | 0.098 | 0.052 |  |
| Occupational complex | | 0.118 | 0.017 |  |
| Annual income status | | -0.010 | 0.844 |  |
| VRS | | -0.052 | 0.290 |  |
| GDS score | | -0.008 | 0.865 |  |
| Smoking status | | 0.289 | <0.001 |  |
| Alcohol status | | 0.152 | 0.002 |  |
| Duration of coffee intake | | 0.494 | <0.001 |  |
| Age of first coffee intake | | -0.236 | <0.001 |  |
| *LCA* lifetime cognitive activity, *VRS* vascular risk score, *GDS* Geriatric depression scale, | | | | |

| **Supplementary Table 2** Results of the multiple logistic regression analyses including the interaction term between lifetime coffee intake and potential covariate(s) predicting Aβ positivity | | |
| --- | --- | --- |
|  | OR (95% CI) ^†^ | *P value* |
| Lifetime coffee intake | 0.102 (<0.001 to 56.639) | 0.479 |
| Age | 1.015 (0.938 to 1.099) | 0.704 |
| Lifetime coffee intake$\times$Age | 1.048 (0.960 to 1.144) | 0.293 |
| Lifetime coffee intake | 0.240 (0.077 to 0.743) | 0.013 |
| Gender | 1.799 (0.587 to 5.508) | 0.304 |
| Lifetime coffee intake$\times$Gender | 1.766 (0.416 to 7.500) | 0.441 |
| Lifetime coffee intake | 0.109 (0.013 to 0.886) | 0.038 |
| Education | 1.010 (0.904 to 1.129) | 0.857 |
| Lifetime coffee intake$\times$Education | 1.096 (0.937 to 1.283) | 0.251 |
| Lifetime coffee intake | 0.434 (0.178 to 1.062) | 0.067 |
| APOE4 | 6.837 (2.925 to 15.980) | <0.001 |
| Lifetime coffee intake$\times$APOE4 | 0.512 (0.126 to 2.073) | 0.348 |
| Lifetime coffee intake | 0.390 (0.156 to 0.973) | 0.044 |
| Clinical diagnosis | 9.708 (3.922 to 24.033) | <0.001 |
| Lifetime coffee intake$\times$Clinical diagnosis | 0.690 (0.175 to 2.718) | 0.595 |
| Lifetime coffee intake | 0.139 (0.009 to 2.099) | 0.154 |
| LCA score | 0.628 (0.288 to 1.368) | 0.242 |
| Lifetime coffee intake$\times$LCA score | 1.473 (0.467 to 4.642) | 0.508 |
| Lifetime coffee intake | 0.420 (0.104 to 1.690) | 0.222 |
| Occupational complexity | 1.153 (0.844 to 1.575) | 0.370 |
| Lifetime coffee intake$\times$Occupational complexity | 0.912 (0.563 to 1.477) | 0.708 |
| Lifetime coffee intake | 0.544 (0.112 to 2.657) | 0.452 |
| Annual income status | 1.269 (0.655 to 2.459) | 0.480 |
| Lifetime coffee intake$\times$Annual income status | 0.704 (0.254 to 1.948) | 0.499 |
| Lifetime coffee intake | 0.247 (0.090 to 0.681) | 0.007 |
| VRS | 0.715 (0.417 to 1.087) | 0.117 |
| Lifetime coffee intake$\times$VRS | 1.341 (0.683 to 2.631) | 0.394 |
| Lifetime coffee intake | 0.221 (0.077 to 0.635) | 0.005 |
| GDS | 0.936 (0.866 to 1.013) | 0.100 |
| Lifetime coffee intake$\times$GDS | 1.063 (0.953 to 1.185) | 0.273 |
| Lifetime coffee intake | 0.241 (0.093 to 0.624) | 0.003 |
| Smoking status | 0.615 (0.230 to 1.648) | 0.334 |
| Lifetime coffee intake$\times$Smoking status | 1.978 (0.585 to 6.690) | 0.272 |
| Lifetime coffee intake | 0.268 (0.101 to 0.710) | 0.008 |
| Alcohol status | 0.557 (0.331 to 0.939) | 0.028 |
| Lifetime coffee intake$\times$Alcohol status | 1.328 (0.584 to 3.017) | 0.499 |
| Lifetime coffee intake | 0.262 (0.087 to 0.789) | 0.017 |
| Age | 1.042 (0.983 to 1.106) | 0.165 |
| Gender | 1.895 (0.627 to 5.723) | 0.257 |
| Lifetime coffee intake$\times$Age$\times$Gender | 1.006 (0.987 to 1.025) | 0.558 |
| Lifetime coffee intake | 0.145 (0.020 to 1.032) | 0.054 |
| Age | 1.042 (0.983 to 1.105) | 0.163 |
| Education | 1.016 (0.910 to 1.136) | 0.774 |
| Lifetime coffee intake$\times$Age$\times$Education | 1.001 (0.999 to 1.003) | 0.360 |
| Lifetime coffee intake | 0.435 (0.179 to 1.059) | 0.067 |
| Age | 1.046 (0.987 to 1.109) | 0.131 |
| APOE4 | 6.846 (2.943 to 15.925) | <0.001 |
| Lifetime coffee intake$\times$Age$\times$APOE4 | 0.991 (0.972 to 1.010) | 0.336 |
| Lifetime coffee intake | 0.232 (0.082 to 0.661) | 0.006 |
| Gender | 1.815 (0.615 to 5.352) | 0.280 |
| Education | 1.029 (0.928 to 1.141) | 0.585 |
| Lifetime coffee intake$\times$Gender$\times$Education | 1.048 (0.955 to 1.150) | 0.321 |
| Lifetime coffee intake | 0.351 (0.164 to 0.752) | 0.007 |
| Gender | 2.274 (0.779 to 6.634) | 0.133 |
| APOE4 | 5.717 (2.729 to 11.975) | <0.001 |
| Lifetime coffee intake$\times$Gender$\times$APOE4 | 0.737 (0.171 to 3.184) | 0.683 |
| Lifetime coffee intake | 0.404 (0.171 to 0.954) | 0.039 |
| Education | 1.065 (0.942 to 1.156) | 0.417 |
| APOE4 | 6.453 (2.822 to 14.757) | <0.001 |
| Lifetime coffee intake$\times$Education$\times$APOE4 | 0.959 (0.863 to 1.067) | 0.445 |
| *Aβ* beta-amyloid, *APOE4* apolipoprotein ε4, *OR* odds ratio, *CI* confidence interval, *LCA* lifetime cognitive activity, *VRS* vascular risk score, *GDS* geriatric depression scale.  ^†^The moderating effects of potential covariate(s) on the relationships between lifetime coffee intake and Aβ positivity were examined by multiple logistic regression analyses including lifetime coffee intake × one or two potential covariate(s) interaction term as well as lifetime coffee intake, and all potential covariates. | | |

| **Supplementary Table 3** Results of multiple logistic regression analyses for assessing the relationships of overall amount of coffee intake with Aβ positivity in non-demented individuals | | | | | | |
| --- | --- | --- | --- | --- | --- | --- |
| Coffee intake | Overall |  | Age of first coffee intake | | | |
|  |  |  | $\leq$55 years |  | $>$55 years |  |
|  | OR (95% CI) | *P value* | OR (95% CI) | *P value* | OR (95% CI) | *P value* |
| Model 1 ^a^ | 0.994 (0.987 to 1.001) | 0.117 | 0.992 (0.984 to 1.000) | 0.057 | 0.979 (0.909 to 1.055) | 0.580 |
| Model 2 ^b^ | 0.995 (0.987 to 1.002) | 0.131 | 0.992 (0.984 to 1.000) | 0.068 | 0.973 (0.896to 1.056) | 0.512 |
| Model 3 ^c^ | 0.991 (0.982 to 1.001) | 0.067 | 0.990 (0.981 to 1.000) | 0.054 | 0.923 (0.795 to 1.072) | 0.294 |
| *Aβ* beta-amyloid, *OR* odds ratio, *CI* confidence interval, *APOE4* apolipoprotein ε4, *LCA* lifetime cognitive activity, *VRS* vascular risk score, *GDS* geriatric depression scale.  ^a^ Adjusted for age, gender, education, apolipoprotein ε4, and clinical diagnosis.  ^b^ Adjusted for covariates in Model 1 plus, LCA score, occupational complexity, annual income status, VRS, GDS score, smoking status, and alcohol status.  ^c^ Adjusted for covariates in Model 2 plus, duration of coffee intake. | | | | | | |
