# Supplementary figures and images for: Coffee intake and decreased amyloid pathology in human brain
Source: Transl Psychiatry. 2019 Oct 22;9:270. doi: 10.1038/s41398-019-0604-5 (PMC6805864; doi:10.1038/s41398-019-0604-5)

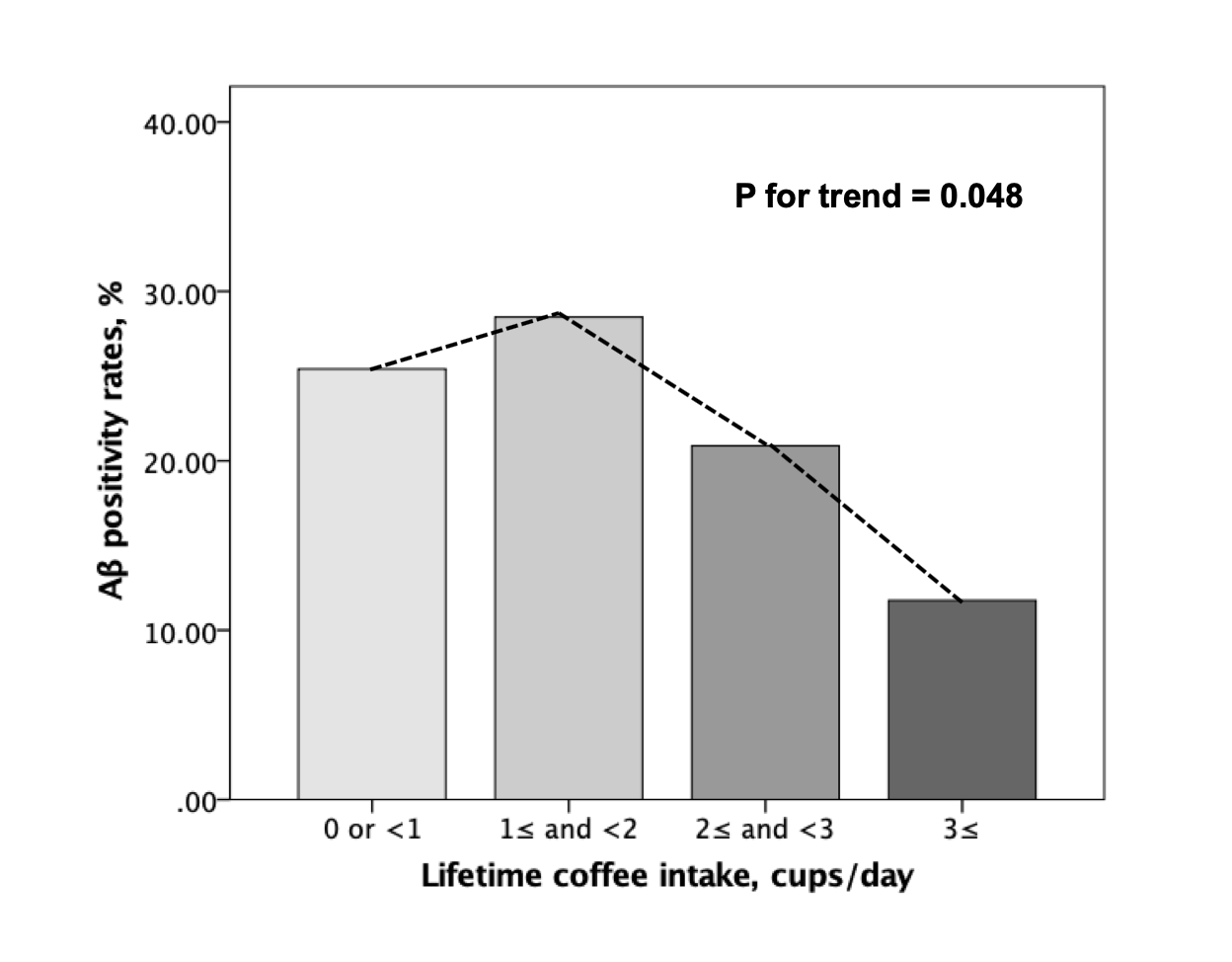

Supplement: Supplementary file 2 — Supplementary Figure 1 [file 41398_2019_604_MOESM2_ESM.tif]
